# ECMO Retrieval over the Mediterranean Sea: Extending Hospital Arms

**DOI:** 10.3390/membranes11030210

**Published:** 2021-03-17

**Authors:** Brianna A. Hildreth, Giovanna Panarello, Gennaro Martucci, Fabio Tuzzolino, Alberto Piacentini, Giovanna Occhipinti, Andrea Giunta, Fabio Genco, Giuseppe M. Raffa, Michele Pilato, Guido Capitanio, Antonio Arcadipane

**Affiliations:** 1 School of Medicine, University of Pittsburgh, Pittsburgh, PA 15213, USA; Hildreth.Brianna@medstudent.pitt.edu; 2 Department of Anesthesia and Intensive Care, IRCCS-ISMETT (Istituto Mediterraneo per i Trapianti e Terapie ad Alta Specializzazione), 90133 Palermo, Italy; gpanarello@ismett.edu (G.P.); apiacentini@ismett.edu (A.P.); gocchipinti@ismett.edu (G.O.); gcapitanio@ismett.edu (G.C.); aarcadipane@ismett.edu (A.A.); 3 Research Office, IRCCS-ISMETT (Istituto Mediterraneo per i Trapianti e Terapie ad Alta Specializzazione), 90133 Palermo, Italy; ftuzzolino@ismett.edu; 4 Perfusion Service, IRCCS-ISMETT (Istituto Mediterraneo per i Trapianti e Terapie ad Alta Specializzazione), 90133 Palermo, Italy; agiunta@ismett.edu; 5 Emergency Rescue Service, 118 Service—Region of Sicily, 91021 Sicily, Italy; gencofabio@gmail.com; 6 Cardiac Surgery Unit, IRCCS-ISMETT (Istituto Mediterraneo per i Trapianti e Terapie ad Alta Specializzazione), 90133 Palermo, Italy; graffa@ismett.edu (G.M.R.); mpilato@ismett.edu (M.P.)

**Keywords:** ARDS, helicopter, transportation, HEMS

## Abstract

The retrieval and transport of patients from peripheral hospitals to high volume extracorporeal membrane oxygenation (ECMO) centers aims to reduce complications and improve survival. In Sicily (Italy), our institute houses a mobile ECMO team that serves a population of around 10 million people for a vast area in southern Italy and Malta. This observational, descriptive study includes all patients that required veno–venous (V-V) ECMO and transport by a mobile team between October 2009 and May 2020. Linear and multiple logistic regressions were applied to explore the risk factors for mortality in the ICU. Kaplan–Meier estimates were generated to predict the survival in patients transported by helicopter or ambulance, and the two cohorts were compared according to their baseline characteristics. Of 122 patients transported, 89 (73%) survived to ICU discharge (50 (41%) patients were transported by ambulance, and 72 (59%) were transported by helicopter). Independent predictive factors associated with mortality in a stepwise multiple regression model were prone positioning, acute kidney injury, and the number of days spent on mechanical ventilation (MV). Kaplan–Meier estimates for survival favored the helicopter cohort (79%) rather than the ambulance cohort (64%). Patients transported by helicopter had better pre-ECMO profiles, with shorter hospital and ICU stays, a shorter duration of MV use, and higher RESP scores, which indicate better survival probabilities. ECMO transport can be carried out safely over long distances; in rural areas with underdeveloped roads, transportation via helicopter or ambulance can extend the arm of the hospital to remote areas. Early ECMO initiation can be crucial in improving survival outcomes, and when transportation is the limiting factor to starting ECMO support, it should be attempted at the earliest logistical stage possible.

## 1. Introduction

Extracorporeal membrane oxygenation (ECMO) is a lifesaving treatment that provides mechanical cardiopulmonary support to critically-ill patients. Often utilized as a last resort in the critically-ill population, it has a progressively improved survival rate compared to that of previous treatments thanks to the technical refining of its materials, better knowledge of the applied management, and the identification of clearer indications considered earlier in the course of the critical disease [1,2,3,4,5]. As a lifesaving treatment, the number of centers practicing ECMO, with various case mixes and volumes, is increasing year by year. However, likely because of its complexity, the clinical outcomes after ECMO placement are better in highly specialized centers with a higher case volume [6,7]. Having designated ECMO centers is essential in delivering safe and efficient care to patients that require this treatment, but this requires the implementation of strategies to also allow patients in regions and communities in remote areas to benefit from this advanced care, since they should not be excluded from a state-of-the-art treatment simply because they live far from an ECMO center and transportation in their critically-ill condition would be too risky [7]. On the other hand, the transportation of patients on ECMO has been controversial given the associated hazards and financial implications, with specific concern for transport over long distances [7,8,9,10,11,12]. Despite the inherent risks, patients have been safely transported to tertiary care centers by specialized transport teams [10,13,14,15,16]. In considering the current need for high volume centers, the transportation of ECMO patients and the establishment of mobile ECMO teams is of paramount importance in ensuring that highly specialized treatment is accessible for underserved rural populations [17]. The feasibility of transportation is one reason for the adoption of this technique worldwide, and in the case of the COVID-19 pandemic, the use of mobile ECMO teams has been observed in many countries [18,19]. To successfully retrieve patients, mobile teams must be experienced in ECMO cannulation, patient and circuit management, and critical care transportation, including transportation by aircraft [7,13]. With safety as the overarching goal, individual hospitals have developed their own specific procedures and protocols regarding transport [20].

In Italy, amidst the H1N1 pandemic in 2009, the Italian National Health Services tasked fourteen medical centers with covering distinct geographical regions in order to treat patients developing severe acute respiratory distress syndrome (ARDS) [21]. During this pandemic (from 2009–2011), adult ECMO cannulation rates increased by 433% in adults worldwide [22]. As a specialized medical center serving a large territory in southern Italy (and also Malta, sporadically), the mobile ECMO team based at the Istituto Mediterraneo per i Trapianti e Terapie ad alta specializzazione (The Mediterranean Institute for Transplantation and Advanced Specialized Therapies) (ISMETT) has performed a number of transports. Infrastructure in this area is limited, with mostly rural terrain present. In addition to the lack of road access, a body of water separates the island of Sicily from the rest of Italy and the surrounding Mediterranean islands (Sardinia and Malta). Both ambulance and helicopter transport play important roles in improving access to highly specialized care. In this manuscript, we describe our institute’s experience in transporting patients on veno-venous (V-V) ECMO for respiratory failure, highlighting the feasibility and safety of the multidisciplinary transportation team, the characteristics of transportation modes (helicopter and ambulance), and the possible geographical extension of ECMO retrievals as a result of the mobile team.

## 2. Materials and Methods

### 2.1. Historical Perspective

ISMETT implemented a heart and lung transplantation program in 2005 [23,24]. As a result, ECMO use was established to provide intraoperative support, a bridge to transplantation, and support to patients in cases of graft failure [25,26,27]. During the H1N1 influenza pandemic, ISMETT was recruited by the Italian ECMO network (recently known as Rete Respira) (a network with a high level of coordination and collaboration with the main ECMO centers in Italy) [28]. A vast geographical region was entrusted to ISMETT, and, given the low infrastructural development of Sicily and the Calabrian regions, the team had to create new protocols to achieve feasible helicopter transportation. Through collaboration with the regional emergency transportation network, real time connections were established with all health care systems in Sicily and other Italian regions.

### 2.2. Transport Protocol

The transportation process includes both a clinical evaluation and a transport plan. Once a referral is made, a senior critical care physician reviews the patient’s ECMO eligibility, medical history, active clinical problems, current therapy, and the feasibility of their safe transportation. Once the decision is made, the mobile ECMO team is deployed, consisting of an anesthesiologist–intensivist, a cardiac surgeon, and a perfusionist. To mitigate the risks of transport, as suggested by many experts in ECMO transportation, a consistent set of supplies and equipment is brought by the ECMO team and a checklist is verified before departure [16]. This checklist includes a centrifugal pump and a console certified and approved for transportation in a helicopter by aeronautical authorities (Cardiohelp–Maquet Getinge Group, Germany) and by the HLS (Maquet Getinge Group, Germany) circuits. Cannulation devices are all brought in twos for each type [29,30]:

- Cannulas of different size: HLS arterial cannulas (15 cm in length) for jugular cannulation, 13, 15, 17, 19, and 21 Fr; femoral venous cannulas (38 cm and 55 cm) 19, 21, 23, and 25 Fr. (Maquet Getinge Group, Germany);
- J-tip guidewires 100 cm and 150 cm in length, currently upgraded with a 180 cm guidewire;
- Various percutaneous insertion kits: a PIK with four multistep dilators 10/12 Fr, 12/14 Fr, 14/16 Fr., 16/18 Fr. (Maquet Getinge Group, Germany); a set of PIK dilator L cannula accessories 18/20 Fr., 20/22 Fr., 22/24 Fr. (Maquet–Getinge Group, Germany); the Opus vascular access kit with a stepped vessel dilator 8/10 Fr., 13 Fr., 16 Fr., 20 Fr., 24 Fr., 26 Fr., and 28 Fr. is also currently available (Medtronic, Minneapolis, MN, USA).

Completing the setup is a portable oxygen tank, surgical instruments, and a complete set of connectors. The following list of requirements is sent to the referring hospital: informed consent to be signed by the patient’s legal representative; two units of cross-matched packed red blood cells to be readily available at the bedside to optimize precannulation hemoglobin if necessary [31]; platelets (if the patient’s platelet count is less than 50,000/µL); a large sterile drape for cannulation; and an ultrasound machine with a linear probe to guide cannulation. Upon arrival at the referring hospital, the team confirms the indications and fully assesses the patient’s condition. Before departure, the following conditions must be fulfilled: the patient’s clinical status must be stable; the patient’s estimated cardiac output must be met (as determined by testing ECMO efficacy tested by increasing revolutions per minute (a “full flow prolonged test”)); and an ambulance with a DC/AC inverter to connect to the ECMO drive unit must be provided. During transportation, all patients are ventilated with a portable mechanical ventilator set to a protective mode and maintaining adequate positive end-espiratory pressure (PEEP).

### 2.3. Modes of Transportation

Ground transport is carried out by ambulance, while air transportation is carried out via rotary wings (Leonardo AW 139 of the Emergency Medical Services of the Region of Sicily). The decision regarding which mode to utilize depends on a variety of factors, including distance, weather, and infrastructural barriers. As a primarily rural Mediterranean region, the island of Sicily is comprised of considerable long distances or winding and hilly roads, which are not suitable for ambulance access. Additionally, a body of water separates Sicily from Calabria (the southern portion of Italy), as well as from the Mediterranean islands of Sardinia and Malta. In these cases, a helicopter would be suitable for transport. Our helicopter includes a complete vital parameter monitor–defibrillator (Physio-Control LifePak 15), a mechanical ventilator (Hamilton T1), a DC/AC inverter to support the ECMO system’s two oxygen sources (for ECMO sweep and mechanical ventilation (MV)), and an aviation-approved rack specifically designated to secure the ECMO system (Maquet-Getinge Cardiohelp), preventing device damage and crew injury.

### 2.4. Patient Cohort

All patients undergoing V-V ECMO transported by our team between October 2009 and May 2020 were included in this study. Their baseline characteristics were recorded prior to ECMO initiation, and were used to calculate predictive scores (Charlson comorbidity index, simplified acute physiology II score [SAPS II], sequential organ failure assessment [SOFA] score, predictive death for severe ARDS on V-V ECMO [PRESERVE] score, Murray, ECMOnet, and respiratory ECMO survival prediction [RESP] score) [28,32,33,34,35,36,37]. The primary variable endpoint considered was survival to ICU discharge. Secondary topics included description of complications occurred during transportation and predictive risk factors associated with mortality. This includes complications that occurred pretransport (during cannulation), during transport, and post-transport (immediately upon arrival at ISMETT).

The distance of helicopter transport was calculated as the difference between the city center in which the referral center was located and ISMETT. To calculate the distance of ambulance travel, the route with the shortest distance between the two hospital coordinates was used. Due to the geographical proximity of ISMETT to two of the referral centers (the Policlinico Palermo P. Giaccone and the Palermo Civico Hospital), 0.5 km was assumed as the average distance. The total mission time was calculated as the difference in time between deployable team departure and patient arrival at our center.

### 2.5. Statistical Analysis

Categorical variables are reported as number and percentage, while continuous variables are reported as mean and standard deviation or median and interquartile range when appropriate. Continuous variables between different modes of transportation were assessed with a T-test or Wilcoxon Rank Sums test when appropriate. Kaplan-Meier estimates were generated to predict the survival function for patients transported with different modes, and statistical differences were assessed with the log rank test. Logistic regression was performed to analyze the association of predictive factors (scores and transportation characteristics) with the mortality event, and odds ratios are reported with CI 95%. A multiple stepwise logistic regression model was also assessed. All analyses were conducted with SAS 9.4 statistical software, and a *p* value < 0.05 was considered the cut-off for statistical significance.

## 3. Results

### 3.1. Patient Characteristics

From October 2009 to May 2020, 122 patients requiring V-V ECMO were transported using our institute’s mobile ECMO team. Their characteristics are summarized in Table 1. Ninety-six patients (78.7%) were male, with a median age of 43 (36–54) years, with a body mass index of 28.0 (24–33) and a PaO_2_/FiO_2_ ratio of 60 (52–67). Looking at the baseline-specific ECMO scores, the median PRESERVE score was 3.7 ± 1.9, and the median RESP score was 1.5 ± 3. Additional baseline pre-ECMO cannulation characteristics included prone positioning for 17 patients (13.9%) and nitric oxide administration for 17 patients (13.9%).

The primary indication for V-V ECMO was acute respiratory distress syndrome (ARDS). ARDS was associated with the following diagnoses: 63 viral pneumonias (H1N1 in 57 cases (90.5%)), 34 bacterial pneumonias, and 2 other acute cases of ARDS. Additional indications for ECMO included 15 traumas, 1 graft failure, 1 pleural empyema, 1 transfusion-related lung injury, 1 tracheal stenosis, 1 case of measles, 1 case of mediastinitis, 1 case of pulmonary fibrosis, and 1 postpneumonectomy complication. The most frequent cannulation strategy utilized was femoro-jugular cannulation (used in 112 cases (91.8%)) and femoro-femoral (used in 10 cases). While supported on ECMO, 65 (53.3%) patients received continuous renal replacement therapy (CRRT). The average number of days spent in the hospital and the ICU were 28 (17–41) and 33 (20–52) days, respectively.

### 3.2. Transport

**Figure 1:**
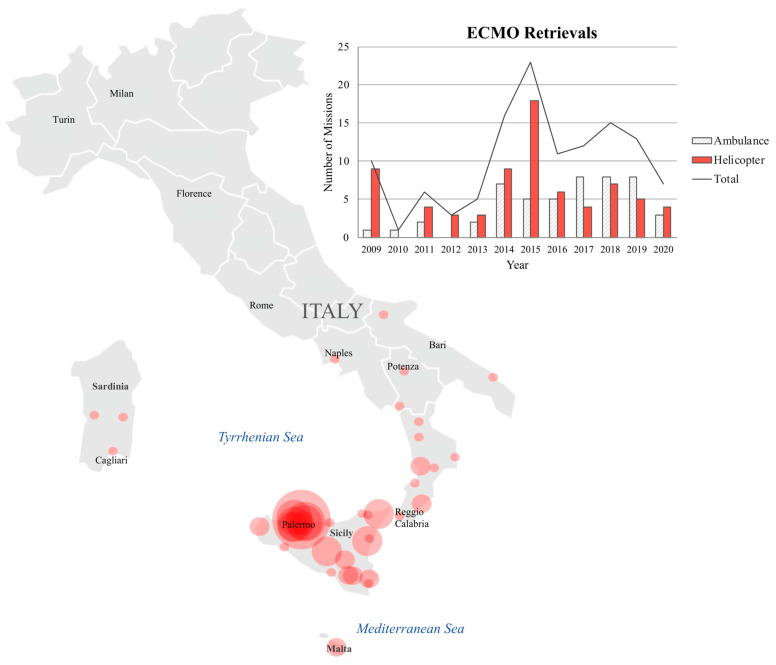
The retrieval map illustrates the geographic distribution of retrievals by referral center, and the bar graph shows the variation in retrieval volume by year. The map was generated using three circles of varying sizes (small, medium, and large), which correspond to the number of retrievals (1–2, 2–3, and 5, respectively). For referral centers with more than five patients retrieved, visual overlay effects were applied. The bar graph depicts retrieval volume by year. White bars represent retrievals by ambulance (*n* = 50), red bars represent retrievals by helicopter (*n* = 72), and the gray line is the yearly retrieval variation for both cohorts combined (*n* = 122).

The average retrieval volume was 10 ± 6 missions per year. (Figure 1) Among all transported patients, 72 (59%) patients were transported by helicopter and 50 (41%) were transported by ambulance. In the ambulance cohort, the median distance transported was 0.5 (0.5–5.9) km (with a minimum distance of 0.5 km and a maximum distance of 67 km) (0.31 (0.31–3.7) miles (with a minimum distance of 0.31 miles and a maximum distance of 41.6 miles)), with a median duration of 3 (2–4) hours. In the helicopter cohort, the median distance travelled was 192.0 (152.7–266.9) km, ranging from a minimum of 72 km to a maximum of 483 km (119 (94.8–165.8) miles, ranging from a minimum of 44.7 miles to a maximum of 300 miles), which includes long distances travelled over sea from the islands of Sardinia and Malta, with a median mission duration of 7 (6–8) hours. The effective median transportation time (the time spent by the patient in the ambulance or in the helicopter) was 10 min (C.I. 7–10) in the ambulance cohort, and 75 min (C.I. 65–120) for the patients transported by helicopter. Combined, patients transported by both modalities travelled a median distance of 140.4 (3.4–200.2) km (87.2 (2.11–124.3) miles), with a median mission duration of 6 h (3–7.5). The map in Figure 1 illustrates the origins of the patients that were retrieved by our mobile team.

Complications (Table 2) occurring during transport included logistical inconveniences, nonfatal complications, and one patient who died during transport. Logistical inconveniences included five missions delayed due to severe weather conditions and four delayed due to helicopter unavailability. Nonfatal complications included two instances of femoral artery lesion, one transport on veno-arterial (V-A) ECMO due to difficult cannulation, one oxygen flow error, one ECMO pump failure, one oxygenator failure, and one power outage in an ambulance. The instance of mortality during transport occurred due to oxygen supply failure while transporting the patient on the helicopter runway.

### 3.3. Survival

**Figure 2:**
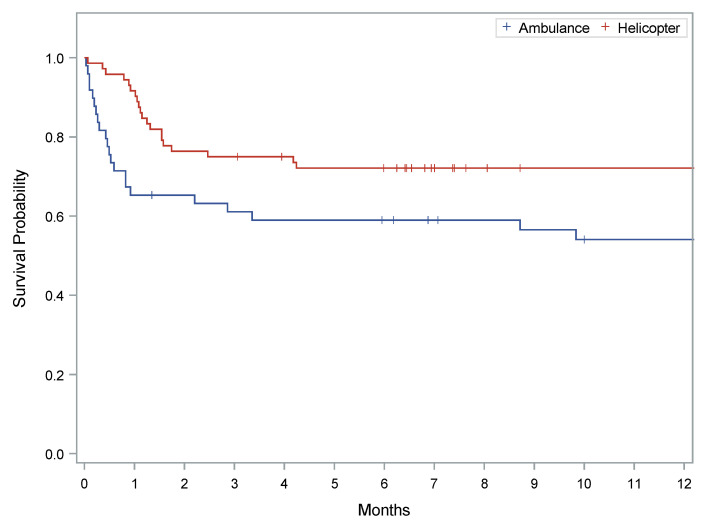
The Kaplan–Meier curve illustrates the survival to ICU discharge rate for patients transported on ECMO. The red line represents patients transported by helicopter, and the blue line represents patients transported by ambulance. Crosses indicate censored data. Kaplan–Meier estimates for survival favored the helicopter cohort (79.2% to ICU discharge) rather than the ambulance cohort (64.0%). A Log rank test was performed, *p* value = 0.03.

Overall, 89 (73%) patients survived to ICU discharge. Following ICU discharge, patients were transferred to either a step-down floor within ISMETT or to an external hospital. As shown by the Kaplan-Meier curve (log-rank test *p* = 0.0251), survival to ICU discharge favored the helicopter cohort (79.2%) rather than the ambulance cohort (64.0%) (Figure 2).

The helicopter cohort had a better pre-ECMO profile, including a decreased length of hospital stay (6.0 (2.0–9.0) vs. 8.5 (4.1–17.0) days, *p* value = 0.0017), a decreased length of ICU stay (3.0 (2.0–6.8) vs. 5.7 (3.0–13.0) days, *p* value = 0.0007), decreased days spent on MV (3.0 (1.5–5.0) vs. 5.1 (3.0–12.5) days, *p* value = 0.0002), higher RESP scores (2.3 ± 2.6 vs. 0.3 ± 3.2, *p* value = 0.0004), and lower ECMOnet scores (5.6 ± 1.6 vs. 6.6 ± 1.4, *p* value = 0.0432). All of these factors are predictive of higher survival probabilities. T-tests performed for additional pre-ECMO predictive factors did not show statistically significant differences between the two cohorts, including in age, BMI, SAPS-II score, SOFA score, Murray score, Charlson score, PRESERVE score, P/F ratio, and creatinine.

Through univariate analysis, the following pre-ECMO values were associated with survival: prone positioning (OR 5.031, *p* value = 0.0031); nitric oxide administration (OR 3.750, *p* value = 0.0142); length of hospital stay (OR 1.050, *p* value = 0.0295); length of ICU stay (1.064, *p* value = 0.0370); and duration of MV use pre-ECMO (OR 1.077, *p* value = 0.0179). Postcannulation factors include AKI (OR 7.800, *p* value 0.0014), CRRT (OR 10.673, *p* value < 0.0001), the number of platelet transfusions (OR 5.000, *p* value = 0.0002), units of packed red blood cells (OR 1.035, *p* value = 0.0164), the duration of ECMO support (OR 1.008, *p* value = 0.0003), the duration of MV use post-ECMO (OR 0.861, *p* value = 0.0006), increased distance transported (OR 0.995, *p* value = 0.0108), and increased mission duration (OR 0.830, *p* value = 0.0265) (Table 3).

In a stepwise multiple regression model, the only factors found to be independently predictive of survival to ICU discharge were AKI (OR 12.958, *p* value = 0.0010), prone positioning pre-ECMO (5.795, *p* value = 0.0112), and days of MV use pre-ECMO (1.090, *p* value = 0.0220).

## 4. Discussion

In this retrospective single-center study, we describe an 11-year experience in transporting V-V ECMO patients over a large Mediterranean island and the surrounding territory, providing a high level of care to remote and underserved areas. The program of transportation was found to be feasible and safe; additionally, we saw better outcomes in patients who, for logistical reasons, were transported by helicopter. It is likely that this group of patients had less severe pre-ECMO severity scores and had been transported earlier to our ECMO hub, with a shorter period of MV use before ECMO.

Our institute occupies a strategic position (from the medical point of view) at the center of the Mediterranean Sea. Aristotle, the ancient Greek philosopher, first described four main winds (Boreas, Notos, Eurus, and Zephyrus) in his meteorological studies, which came eventually to denote the cardinal directions on an ancient compass, with a wind rose ideally positioned at the center of the Mediterranean Sea to also denote geographical directions in navigation. Considering that our institute is at the center of the wind rose, we organized helicopter medical service (HEMS) at 360° degrees, bearing an interfacility arm protruding above the Mediterranean Sea, offering deployable ECMO services within an ideal radius above 500 km, and encompassing other regions such as Sardinia (another large Mediterranean island) and other countries (Malta and North Africa).

The decision to transfer an unstable patient, though historically defined in the classic study by Erenwerth focalizing on “risk class patients”, can still be a difficult decision today [38]. Many factors can jeopardize the outcomes and success of a safe transport. We can distinguish clinical problems (such as patient condition, hemodynamic instability, and need for ventilation), from logistical problems, such as the weight of the equipment, device airworthiness, and flight stressors, the latter being a typical subject of aviation medicine affecting both the patient and the medical crew. In our case series, the logistical problems were rare because we relied on a public rescue transport able to interact with all of the players of the public health care system. A complete connection with the regional emergency transport system by helicopter assures a rapid and effective interdependence of our local teams, but since our helicopter is the only one available in the region, when a call is managed by a referral center, the referring ECMO center usually agrees to set up the mission as soon the availability of the means of transportation permits, since it may be unavailable in the case of an emergency mission.

Considering the past, we will not address all the problems related to safety of flight, weather, endurance, and the speed of aero mobiles, which could be the subject of a different analysis. To better give an idea about device weight, the first portable defibrillator was invented in Belfast by F. Pantridge in the mid-1960s [39]. This edition weighed 70 kg and was powered by car batteries. The device was “portable” in the sense it could be delivered by an ambulance, but could not be unloaded. Modern defibrillators weigh ten times less than original ones. In 1953, Dr. J.H. Gibbon used the first heart–lung machine in humans undergoing cardiac surgery. In 1972, Dr. Donald Hill treated an adult with the device. Dr. Robert Bartlett developed modifications in order to support the heart and lungs for a prolonged period of time, and used it to treat the first newborn in 1975, in Irvine, California [40]. The continual development of ECMO (also being developed by Dr. Theodor Kolobow) has enabled the program to expand from pediatric populations to adults. The current weight of our portable equipment (Cardiohelp, Maquet-Getinge Group, Germany) is 10 kg.

From another historical point of view, the transfer of critically ill patients between hospitals was developed mainly for severe trauma patients, where centralization to “hub” hospitals with large volumes and continual experience saw a lower mortality for this group of patients [41,42]. The data here, extracted by our single center experience over a period of ten years of a deployable ECMO team, seems to suggest that the early transfer of a patient under extracorporeal circulation in a “spoke to hub” fashion, together with a properly experienced medical crew, can be performed safely even over long distances [43]. Early ECMO availability via fast dispatch and initiation timing (time “0”) can be crucial for improving survival [14]. This was one of the main results of the EOLIA trial [44]. Interestingly, in our case, the final decision to transfer a patient by ground or by air (rotary, or fixed wing) was not determined by general guidelines, while local orography factors can affect final transportation modality decisions. In this sense, the criteria for transportation that we have adopted may not be universally adoptable. Many of the patient transfers have been between regions, with Sicily being the largest island region in Italy and in the Mediterranean area [45]. 

We found that associations among early dispatch (associated with a better pre-ECMO profile) and early ECMO team deployment via rotary wing versus ground vehicle resulted in a shorter hospitalization period, a shorter duration of need for MV support, and a higher RESP score, which should indicate a higher level of survival in this class of patients.

In conclusion, in this era of multiple infectious disease outbreaks, ECMO preparedness is of utmost importance [17,46,47]. The transportation of patients under ECMO support in helicopter is feasible, but it is important to document hospital-specific protocols and procedures to inscribe the ECMO transportation in a wide framework of critically-ill patients. The experience cumulated across 11 years of ECMO transportation will likely reveal itself to be of paramount importance during the COVID-19 pandemic in our region.

## Figures and Tables

**Table 1:** Baseline characteristics, pre-ECMO profile, diagnosis, and treatment data.

**Patient Characteristics**	
Age (years)	43 (36–54)
Male gender	96 (78.69)
Weight (kg)	82 (70–96)
Height (cm)	170 (165–175)
BMI (kg/m^2^)	28.0 (24.4–33.0)
**Pre-ECMO Profile**	
Hospital length of stay (days)	6.2 (3.0–12.1)
ICU length of stay (days)	4.0 (2.0–9.0)
Days on mechanical ventilation	3.0 (2.0–8.0)
Prone positioning	17 (14.05)
Nitric oxide	17 (14.05)
PaO_2_/FiO_2_	60 (52–67)
SAPS II	39 (31–46)
SOFA score	8 (6–10)
Murray score	3.5 (3.5–3.75)
PRESERVE score	4 (3–5)
ECMOnet	5.5 (5.0–7.0)
RESP Score	2 (0–4)
Charlson comorbidity index	1 (0–2)
**Treatment Data**	
Diagnosis	
Viral pneumonia	63 (51.63)
Bacterial pneumonia	34 (27.87)
Trauma	15 (12.30)
Other acute respiratory diagnosis	7 (5.74)
Other chronic respiratory diagnosis	2 (1.64)
Graft failure	1 (0.82)
Drainage	24 (23–25)
RBC volume (mL)	1793 (750–3672)
RBC units	7 (3–15)
FFP	900 (500–1750)
Platelets	880 (300–2071)
Hospital length of stay (days)	33.0 (20.0–52.0)
ICU length of stay (days)	28.0 (17.0–41.0)

BMI: body mass index; ICU: intensive care unit; SAPS II: simplified acute physiology II score; SOFA score: sequential organ failure assessment; PRESERVE score: predictive death for severe ARDS on V-V ECMO; RESP Score: respiratory ECMO survival prediction score; RBC: red blood cells; FFP: fresh frozen plasma.

**Table 2:** Rescue mission characteristics and complications.

**Rescue Mission**	
Transport distance km (miles)	140.4 (87.2) (3.4–200.2) (2.11–124.3)
Transport duration (hours)	6.0 (3.0–7.5)
Helicopter	72 (59.01%)
Ambulance	50 (40.98%)
**Complications**	
Delays due to helicopter unavailability	4 (3.3%)
Femoral artery lesion	2 (1.6%)
Transport on V–A ECMO due to difficult cannulation	1 (0.8%)
Oxygen flow error	1 (0.8%)
Pump failure	1 (0.8%)
Oxygenator failure	1 (0.8%)
Power outage	1 (0.8%)
Mortality during transport	1 (0.8%)

**Table 3:** Logistic regression of the association between predictive factors and mortality.

		95%	CI	
	OR	Lower	Upper	*p* Value
Age (years)	1.001	0.97	1.033	0.9547
Sex (female)	1.729	0.593	5.042	0.3157
Weight (kg)	0.993	0.975	1.011	0.4128
Height (cm)	1.016	0.974	1.059	0.4656
BMI (kg/m^2^)	0.959	0.9	1.022	0.1988
Prone	5.031	1.724	14.684	0.0003
Nitric oxide	3.75	1.304	10.78	0.0142
Length of hospital stay pre-ECMO	1.05	1.005	1.096	0.0295
Length of ICU stay pre-ECMO	1.064	1.004	1.128	0.037
MV days pre-ECMO	1.077	1.013	1.146	0.0179
P/F	1.006	0.975	1.037	0.7173
Drainage	0.857	0.675	1.087	0.2037
SAPS II	1.037	1	1.076	0.488
SOFA	1.056	0.928	1.203	0.4057
Murray	0.261	0.0049	1.375	0.1131
PRESERVE	1.153	0.935	1.421	0.1842
ECMOnet	1.205	0.832	1.744	0.3231
RESP score	0.9	0.79	1.026	0.1142
Charlson	1.146	0.874	1.502	0.3235
RBC units	1.035	1.006	1.064	0.0164
FFP	1.411	0.444	4.486	0.5598
Total FFP (yes or no)	1	0.998	1.001	0.5781
Platelets	5	2.123	11.775	0.0002
Total platelets (yes or no)	1.001	1	1.001	0.0387
Transport distance (km)	0.995	0.991	0.999	0.0108
Transport duration (hours)	0.83	0.704	0.978	0.0265
AKI	7.8	2.216	27.457	0.0014
Furosemide	1.755	0.725	4.248	0.2124
CRRT	10.673	3.455	32.97	<0.001
Creatinine	1.048	0.833	1.318	0.6902
MVD post	0.861	0.791	0.938	0.0006
Duration of ECMO support (days)	1.008	1.004	1.012	0.0003

## Data Availability

The dataset used and analyzed are available from the corresponding author on reasonable request.
